# Cocreating guide dog partnerships: dog training and interdependence in 1930s America

**DOI:** 10.1136/medhum-2018-011626

**Published:** 2019-02-28

**Authors:** Neil Pemberton

**Affiliations:** CHSTM, University of Manchester, Manchester M13 9PL, UK

**Keywords:** performance, anthropology, history, medical humanities, cultural history

## Abstract

This article scrutinises issues around disability and dependent (interdependent) agency, extending these to non-human animals and service dogs, with a sustained reference to the training of guide dogs. It does this through a detailed engagement with the training methodology and philosophy of The Seeing Eye guide dog school in the 1930s, exploring the physical, bodily and instrumental means through which the guide dog partnership, and the identity of the instructor, the guide dog and the guide dog owner, jointly came into being. The novelty of the article lies in how it reconsiders what interdependence meant and means from the perspectives drawing from historical and sociological literature on dog training. In doing so it opens up new ways of thinking about service animals that recognise their historical contingency and the complex processes at work in the creation and development of interdependent agency.

This article has two central aims. First, it aims to explore the issues around disability and dependent (interdependent) agency, extending these to non-human animals and service dogs, with a sustained reference to the training of guide dogs. I will do this through a historical exploration of the training methodology and philosophy of The Seeing Eye guide dog school during the 1930s, exploring the role of the instructor and the nature of the training of humans and dogs. The other aim, with which I will begin, is to use this reconstruction of a historically situated instance of dependent agency to show the value of reconsidering what the interdependence of dog training has meant, historically and sociologically.

## Introduction

In recent years, disability theorists have explored the limits of the social model of disability, which over the last three decades has challenged the medical model’s conceptualisation of disability as an individual ‘problem’ resulting from a biological impairment.1 Exponents of the social model of disability argue that it is society’s environments—both physical and social—that render disablement, an affirmation that opened possibilities for activism and change. Yet, while acknowledging the achievements of the social model of disability, disability theorists are nonetheless concerned with the limits of a rights-based model that exalts ‘independence’ as the main route to a dignified life and privileges the autonomous individual. This social model, as Eva Feder Kittay observes, ‘stigmatizes the lives of those whose impairment requires a carer for the disabled person to live out their life, but also, at the same time, denigrates the care and the carer’.2 Kittay’s perspective draws upon and extends feminist ethics of care that prioritise the obligation of caring for others and the dependence we all have on caring relationships.3 A number of disability theorists are now increasingly questioning the notion of the independent, rational individual in an attempt to destabilise and deconstruct ‘the fantasy of ableist human one-ness’ and of dependence as undesirable and a failure of the self, arguing that ‘Disability demands mutuality, support and interdependence’.4 Some dependencies involve care tasks that require subtle and complex co-choreography by carers and recipients, connecting ‘ontologically and haptically through their differences’ to forge what Akemi Nishida has usefully termed ‘affective relationality’.5

More broadly speaking, recognising dependence as an essential state of being human, experienced by all individuals over the course of their lives, destabilises ‘able-ness’ as much as ‘dis-ableness’. We are all, disabled and able alike, so situated in complex systems of interdependence and dependence that the idea of individual agency, to a certain extent, is nothing more than an illusion. Action and identity are always negotiated and enacted through ‘dependent agency’ and not rooted in any one body but dispersed.6 The implications of dependent agency are adeptly illustrated in Hélène Mialet’s Actor-Network-Theory informed ethnography of the celebrated physicist Stephen Hawking. For Mialet, his disability makes legible what normally passes unnoticed: the complex network(s) of interdependence between humans and non-humans every person requires to function.7 Hawking’s carers, assistants, students, voice technology and technicians—among other elements—constitute his ‘extended body’, over which his competencies are distributed. Mialet’s analysis destabilises the myth of the independent rational individual, especially that of the scientist.8

Significantly, the importance of interdependence is increasingly being acknowledged in another area of critical enquiry: animal studies. This interdisciplinary field has challenged the commonsense notion that neatly divides humans from other living beings, cloaking the degree to which human and animal lives are interdependent with those of other species. Donna Haraway has called for narratives about ‘co-habitation, co-evolution, and embodied cross-species sociality’ to be recovered in order to overturn narratives of human domination and the disciplining of animals.9 Stories about multispecies interdependence, moreover, operate on multiple levels, from the microscopic organisms throughout the mammalian body, to the macroscopic level, where we meet my subject, the irreducible guide dog–human unit. The guide dog partnership involves interspecies encounters that create the potential for synchronisation and communication across species boundaries, a phenomenon that Vinciane Despret has termed ‘anthropo-zoo-genesis’.10 Despret’s foregrounding of the affectivity of certain kinds of practices enables us to explore how animals and human become attuned, and the conditions under which each party becomes more interesting to the other.

Despite a common interest in relationality and interdependence, animal studies and disability studies have only recently begun to explore the interpretative possibilities of potential coalitions between these two related but diverse fields. Of course, sound reasons exist for dialogue between these fields to be potentially fraught, not least because of the long-standing oppressive, ableist impulse to rank some people with disabilities alongside non-human animals, which helps explain some of the reluctance of disability scholars to destabilise the category of ‘human’. Sunaura Taylor has called for solidarity between animal and disability rights to overturn the long-established association of dependence with being a burden, as this misleading and stigmatising association drives discrimination and abuse of both human and non-human animals.11 Taylor’s rights-based account considers the possibilities created by a focus on mutual dependence in the final sentence of the chapter entitled ‘The Service Dog’. Describing herself and her service dog Bailey, who had also become disabled, she writes compellingly of two ‘vulnerable, interdependent beings of different species learning to understand what the other needs. Awkwardly and imperfectly, we care for each other’.12

Taking Taylor’s final evocative remark about mutual dependence as a useful starting point, this article offers new ways to forge links between the fields of disability studies and animal studies through a historically situated and empirical analysis of a specific relationship often extolled as an exemplary model of interdependence: the guide dog partnership. Early sociological explorations tended to present the guide dog in highly utilitarian and objectified terms; these assumptions can still be found in professional literature on rehabilitation, policy circles and wider public discourse.13 Rejecting such a view, the self-avowedly posthumanist literary scholar Cary Wolfe has described the guide dog as no mere prosthetic with which to integrate disabled people into mainstream society, but rather as ‘an irreducibly different and unique form of subjectivity…constituted by complex relations of trust, respect, dependence and communication’.14 In a similar fashion, cultural and historical geographer Philip Howell has singled out the guide dog partnership as a model of ‘dependent agency’. As the human component requires training alongside their animal assistant, ‘that agency can in no way be equated with autonomy – here the agency of the (disabled) human is dependent in large part upon the agency of the animal’.15

My understanding of the guide dog partnership is informed by Wolfe and Howell’s suggestive characterisation of service animals as models of dependent agency, and guide dog partnerships as highly mutualistic and dependent relationships. However, in this essay I attempt to approach dependent agency through the lens of guide dog training, exploring in this context what interdependence means, and might mean, by examining the practices and discourses mobilised in the process. I focus on the training methodologies developed by The Seeing Eye in the 1930s: America’s first and oldest guide dog school. My core focus on dog training marks a departure from existing histories of guide dogs which have primarily focused either on pioneers of the guide dog movement or the shifting paradigms of disability within which the movement became established.16

My aim in extending this existing work is not to consider dependent agency or guide dog training solely in terms of disability studies or the history of disability, but rather to reappraise it through perspectives from the history and sociology of dog training. Three main points from these discussions have informed my recontextualisation of dependent agency. First, as Justyna Wlodarczyk has ably demonstrated, dog training is a socially, culturally and historically constituted set of practices and discourses, informed by historically shifting understandings of the dog–human relationship.17 Second, broader political and social developments both stimulate and limit the development and institutionalisation of dog training cultures, as Chris Pearson has shown in his considered work on the development of police dogs in Paris at the turn of the 20th century.18 Third, while the intersubjective process of dog training shapes both human and canine minds, feelings and bodily expressions, affinities, attunements, and connections of embodiment and intercorporeality made across species may only be partial.19 Natalie Hannah Porter’s multispecies ethnographic work is important here, as she focuses on the constitution of care practices in the kennelling and training of rescue dogs to reconsider well-being as a trans-species phenomenon, highlighting the challenges in developing caring relationships with dogs whose behaviour is regarded as an ‘inconvenience’ to humans.20

In light of these concerns, this article historicises dependent agency as articulated and enacted in the training discourses and practices developed by The Seeing Eye in 1930s America. In doing this, critical attention is given to the figure of the instructor, to whom The Seeing Eye devolved responsibility for bringing the guide dog partnership into being. As this article will demonstrate, The Seeing Eye conception of a successfully functioning guide dog partnership was inextricably dependent on the instructor’s meticulous cultivation and management of intersubjective relationships, cross-species companionship and communication. This entailed the creation of novel training practices promoting complex attunements and connections of embodiment between dissimilar living beings: two bodies with diverse capacities learning to tailor themselves to each other.21 Where appropriate in my analysis, I acknowledge the limits of training practice: as much as there was potential for attunement between the species, there was also prospective disconnection and dissonance in the making of the guide dog partnership. I also highlight some of the cultural stakes and tensions underpinning this training methodology.

To explore the labour involved in the production of dependent agency, I draw upon the Hubbard Lectures—also known as The Gospels of The Seeing Eye—compiled in 1938 and consisting of 3000 pages in 20 volumes. To my knowledge, these sources have yet to receive academic attention.22 The unpublished transcripts of lectures by the head trainer at The Seeing Eye, Elliot S (‘Jack’) Humphrey, lay out the affective practices and conceptual underpinnings of guide dog training in meticulous detail and are a rare anomaly: animal training has tended to be a practical, highly performative and orally transmitted craft.

## The Seeing Eye and a declaration of independence

While there exists significant documentary evidence attesting to a long tradition of people with visual impairment using dogs to help with orientation, it was not until the early decades of the 20th century that guide dog training became professionalised and institutionalised. In the closing years of the First World War, soldiers permanently disabled by injury and infection returned home in significant numbers, their impairments a living testament to the horrors of war. The use of chemical weapons on the front made blindness one of the more serious corporeal consequences of war, prompting experiments in the rehabilitation of ‘war blinded’ solders across the world.23 While these programmes were explicitly intended as ‘rehabilitation’—to reshape ex-servicemen into ‘productive’ members of industrialised and urban economies—they were also intended to save former soldiers from ‘stigmatising’ relationships of dependence, returning them to a life of ‘respectability’ and ‘productivity’. Emerging at an important juncture in attitudes towards disability and social policy, the initial guide dog movement may be aligned with a host of rehabilitative institutions that sprung up in the First World War and its aftermath.

It was in Germany—a country with a successful history of canine training—where dogs were first enrolled in the rehabilitation of wounded soldiers: the German government’s war-blinded soldiers’ association and the Society of the German Shepherd Dog formed a coalition to train dogs and people with visual impairments to walk together. Help for people with visual impairment had previously been left to charitable philanthropy, but it was now believed the public owed a debt to the ex-servicemen blinded while defending their country. The war had demonstrated the breadth of roles dogs could undertake: canine couriers, rescuers and sentries had also provided companionship and comfort to soldiers struggling with the hardships of life in the trenches. It is little wonder some historians have emphasised the significance of the First World War’s militarisation of the human–dog bond as an ‘interspecies moment’.24

As newspapers across the world publicised the training of dogs to guide ex-servicemen, activists, voluntary associations and private philanthropists expanded the scope of the guide dog movement to include civilians. In 1929, the philanthropist and dog breeder, Dorothy Harris Eustis, and salesman Morris Frank, who had lost his sights as a teenager, established The Seeing Eye guide dog school. The first of its kind in the USA, the school was based in Nashville for several years before moving to Morristown in New Jersey.25 Testimonials announcing that guide dogs provided their human partners with independence became ubiquitous in US public discourse: the idea of an autonomous, independent subject was central to American ideals of citizenship and The Seeing Eye ethos. In 1929, Morris, who travelled across the USA with his guide dog to demonstrate his independence, famously described his new found freedom in these terms: ‘I have signed my Declaration of Independence and enjoy it to the fullest with my dog Buddy’.26

Working with a dog allowed guide dog owners more independence, yet they are only independent to the extent that they were less reliant on human people. In this context, self-determination as measured by independence from the necessary help and caprices of other humans was considered an essential component of a full life. While the belief that charity made people dependent was certainly not a novel idea, the ideology of liberal individualism intensified during the Depression, alongside the associated values of self-help and sacrifice.27 Rather than mounting a public defence of dependence, The Seeing Eye proposed a new solution, one rooted in the discourse of rehabilitation in the same way the German guide dog movement sought to rehabilitate and return soldiers’ bodies to ‘productive’ civic and economic citizenship: a blind person’s quality of life was determined by the ‘freedom’ and ‘independence’ guide dogs could provide.

The Seeing Eye’s initial choice of canine—the German shepherd—was not unproblematic: the breed was popularly believed to be a direct descendent of the wolf and had an established reputation in the USA as a ‘police dog’.28 American breeders had imported German shepherds since the 1910s, and The German Shepherd Dog Club of America, inspired by similar exhibitions in Germany, organised displays of protection work, such as defence of an owner during a mock attack. The philosophy and methodology of the renowned German police and military dog trainer, Konrad Most, instilled fear in his dogs to foster canine submission and unconditional obedience. For Most, training was a physical struggle, much like the taming of a wild animal, and required (masculine) firmness, strength and courage. For its American enthusiasts, the German shepherd’s supposed 'wildness' served as a way of conquering nature and a means of revitalising urban masculinity.29

To prepare German shepherds for guiding necessitated the development of a new kind of dog–human relationship, unrecognisable from the one presented in the dog training methodology and philosophy centred on the German shepherd and service dogs at that time. The guide dog was expected to take the lead, always walking ahead of their human partner, and in contrast to the complete subservience required of protection dogs, it was essential for a guide dog to disobey commands that might bring the partnership into danger: what The Seeing Eye memorably termed ‘intelligent disobedience’.30 The creation of this new dog–human relationship was believed to rest almost entirely on the forging of a positive emotional attachment between the handler and the dog. At every stage of training the affective dimensions of this relationship had to be s carefully managed; for, it was believed, a dog’s capacity for both obedience and disobedience was contingent on their emotional investment in their handler as a companion who would meet their needs.

## Caress

At the heart of The Seeing Eye ethos, exemplified in Jack Humphrey’s lecture notes, was the belief that dogs could not be forced to enter the relationship required for successful guiding. Before any training could take place at the school’s campus, a dog had to be assured that their instructor was a ‘companion’ they could depend on to meet their needs. The first week was therefore devoted to the instructor demonstrating they were dependable: a ‘friend’ who would be ‘fair’ and ‘not fail in a pinch’:31

To do this you must arrange the first work in such a manner that everything is pleasant for the dog. Notice that I have not said “so that everything is pleasant for you” but for the dog. During this time you will play with the dog, romp with it, rub it, scratch it, feed it and clean it. In other words, you will do everything possible to prove to the dog that you are a real companion in whose company it may be joyful and in whose company it may give free rein to the willingness to please which is inherent in most dogs.32

Thus, the idea was not ‘to teach the dog to act as a guide but to teach it that you are a friend, to teach it your odor, your voice and your hand caress’. Time and space were provided for the instructor and the dog to establish familiarity and begin to acknowledge each other as companions. To overcome the challenges of working together in relationship lacking a common verbal language, the instructor had to attune themselves to the dog’s distinctive ways of making an acquaintance and their species specific sensorial, affective and tactile world.

To illustrate this further, we can turn to a series of photographic tableaus created to accompany the lecture notes, depicting the right and wrong ways to ‘caress’ a dog. The body areas where dogs tend to welcome and consent to physical contact are shown, such as behind the ears and the chest, along with correct touching methods. The head, for example, should be rubbed, not patted or hugged. The underlying logic here was that these standardised touching practices would build tactile familiarity, believed to be of such importance that all grooming was delayed until there was no doubt the dog had become tolerant of their trainer’s touch. It is important to emphasise that these lecture notes were presented by Humphrey as guidelines rather than gospel: a trainer was expected to learn and adapt to the specific tactile preferences and aversions of their dog, and then, to facilitate communication and training possibilities, incorporate these into their training practices. Thus, instructors learnt the core of The Seeing Eye training philosophy: the human partner was obliged to adapt to the specificity of an individual dog.

In order to communicate across species lines and create companionship through a labour-intensive programme of training, instructors were required to mobilise and instrumentalise caress both manually and verbally.33 Speech had to be imbued with tactile and affective qualities that were both interesting and understandable to their canine coworkers: in other words, instructors had to learn to stroke their canine coworkers with words.

Just as with a physical caress, there were strict guidelines for articulating each ‘verbal caress’. *Atta-girl*, used to reward work well done, should be delivered in a ‘happy and appreciative’ tone, and at a low pitch to indicate ‘calmness, surety and friendliness’: a high pitch exuded excitement, fear or anger.34 Significantly, caressing tones were not limited to verbal rewards. The directional commands, such as *right*, *left* and *forward*, also required specific caressing qualities rather than authoritative, harsh tones. As Humphrey explained, this enabled the dog to *feel* the command, not as a demand for absolute obedience, but as a request their trainer trusted them to comply with if it was safe to do so.35 The instructional regimen presented caresses as a form of ‘pay cheques’, units of exchange to give recognition to the dog for an effective thought or safe decision. In the world of The Seeing Eye, caress gave the dog a stake in the training and the partnership itself.

Articulating a caress, however, was considered no easy task. An absence of suitable and expectant caressing tones, it was feared, could imperil both a trainee dog’s cooperation and the companionship between trainer and trainee, as Humphrey explained:

If your “That’s the girl” is simply mechanical and your thoughts are a hundred miles away or if your verbal reward is given as though you were saying, “You son of a gun,” or even if your thoughts are right there but you do not really appreciate what the dog has done; then the dog will take your verbal expression at its exact value, and will think “He does like me”…Be careful, Do not let your “that’s the girl” become automatic.

A responsive instructor therefore had to become more aware of their own emotional registers and gain self-mastery of their body and emotions, including their temper:

I can truthfully say that with every instructor we have had, I have seen the results of days, months, of careful education ruined through a momentary loss of self-control. That one moment may upset everything you have done up to that time, through breaking down the dog’s confidence. You can write this down and paste it in your hat: before you can control the pupil, you must learn to control yourself. If you get can get that, you are already half way toward being an instructor.36

Thus, an instructor’s emotional discipline was of paramount importance in managing the training relationship and companionship with their canine coworker.

With these quotations in mind, we can make several points concerning the emotional interdependence deemed essential to the production of dependent agency in The Seeing Eye world. First, dogs were understood not as ‘machines’ but as complex, sentient creatures, whose feelings no instructor could afford to ignore. This ascription of agency went even further: dogs were said to be masters of reading human emotions. While Humphrey believed emotions to be shared across species—a belief that historians of emotions have argued was established in the post-Darwin period—he was also aware of the significant physiological differences, which, if not properly understood, were potential sources of emotional and communicative disequilibrium.37 ‘The dog’s [sense of hearing]’, he explained, ‘is more discriminating in judging the tone of voice than is the average human being’.38 The ability to detect subtle variations in tone in the human voice meant a dog could easily pick up unintentional cues; the verbal caress therefore required concerted effort from the canine’s human coworker.

In order to manage and train their own emotions, instructors had to incorporate a sense of canine sensibilities into their bodily performance. In this light, caress constituted a shared, cocreated affect registering both human bodily action and the dog’s perceived aptitude for reading human emotions: a prime example of Despret’s ‘anthropo-zoo-genesis’.39 The practice of caress rested on the explicit and implicit blurring of the boundaries between nature and culture, gender and species. In The Seeing Eye world, guide dogs were always a ‘she’, the instructor always a ‘he’: instructors not only believed female German shepherds were less aggressive and easier to train than males, but that they possessed innate nurturing qualities and were more responsive to caress. In wider public discourse seeing eye dogs were often compared with human ‘wives’, ‘girlfriends’ and ‘mothers’. The dependent relationship to be cultivated was understood as entirely natural: in keeping with heterosexual gender norms and expectations. In a further elaboration of these gender ideals, the training of female dogs required a combination of physical stamina and emotional discipline: traits firmly embedded in the masculine sphere. Instructors worked with a dominant masculine discourse of human emotional restraint set against the perceived uncontrolled emotion of human feminised bodies.

The role of corrections in guide dog training provides a further arena for gauging the challenges and tensions in creating dependent agency and managing emotional interdependence. Although trainers stressed the need for cooperation, power relations between the dog and the handler were never free from hierarchy. Just as it was necessary to reward a trainee dog, it was equally necessary to signal undesirable behaviour and decisions. The rules governing the expression of corrections were framed within the same mode of emotional intersubjectivity that guided the language of verbal caresses. Thus, if a joyful *Atta-girl* was the pure embodiment of positive affect, *Pfui-Pfui* was its corollary opposite, embodying negative affect to signify to the dog ‘displeasure at the work being done’.40 Whereas *Atta-girl* required an uplifting and pleasant tone, *Pfui-Pfui* demanded a ‘rather harsh’ tone, while the pitch needed to be high—though not too high—to relay the ‘anger felt’.41 The less displeased *Unh-Unh* required a lighter, subtler corrective tone and a less harsh pitch. However, there was a profound problem with any correction: it channelled negative affect through the trainer’s voice, and, to use Humphrey’s phrasing, constituted ‘a force that drives away the animal’.42 Corrections thus had to be carefully used: an instructor who ‘punished’ was operating without reasonable restraint and cared little for a dog’s feelings or presenting the situation in a manner the animal could understand. As soon as possible after a correction had been made, the instructor was expected to execute an exercise the dog could perform easily and therefore receive caress: ‘to put back the willingness you draw out with your correction’.43 In the world of The Seeing Eye, the instructor had to quickly remind the dog that ‘master’ was always fair and affectionate, thus maintaining cooperation.

## ‘A Month of Blindness’

In addition to embodying a set of emotions to which a dog could read and respond, instructors were expected to fashion a body that could communicate to the dog the requirements of a future handler with visual impairment. This meant that every step the instructor took with their trainee dog, along with their reactions, orientation, bodily positioning and even their speed of walking, had to anticipate those of the future handler.44 The instructor’s bodily simulation of the future handler further illustrates how dogs were perceived in The Seeing Eye world as highly responsive beings capable of reading and, more significantly, being affected by human communication cues. To grasp the point of view of the future handler, understand the contours of their world and adjust to a sensorial world in ways that did not always privilege vision, instructors underwent an obligatory point of passage: they were blindfolded 24 hours a day for the first month of their training. This perceptual reorientation enabled the instructor to accumulate tacit knowledge about working with a guide dog from the perspective of a visually impaired handler and the capacities that could be drawn upon to manage and monitor their own body’s affective potential. ‘At the end of that period’, elaborated Humphrey, ‘you should understand, not only the feel of your dog and the interpretations of her signals, but you should also realize the actions or reactions of a sightless master under varying working conditions’.45 The month of blindness served as a mechanism of estrangement, through which the instructor could escape their habituation to the seeing world and therefore prevent the projection of sighted priorities, assumptions and cues into the contact zone, undermining the capacity of the future dyad.

To fulfil this crucial objective, and once the blindfold had been removed, Humphrey advised his instructors never to pre-empt a fault that they could ‘see’, as to do so made the dog unsafe for a handler with visual impairment.46 His golden rule was as follows: ‘You must remember that the dog leads and you follow, and remember that this must be the manner of working – the dog leading and the man following…even if it means taking a bump’. Thus, if the dog failed to stop and went over a curb, the instructor must stumble as the future handler might. Likewise, if the dog passed too close to a pedestrian, the instructor must not turn their shoulder or attempt to change a dog’s course but actually collide with the pedestrian.47 Turning to avoid a bump, it was believed, could signal to the dog that they should react to the body motion communicated through the change in harness tension, rather than the object to be avoided.

By submerging cues from their sighted subjectivity, and signalling the unpleasantness of bumping into or stumbling over obstacles, the instructor was prompting the dog to take ‘responsibility’ for their human coworker. As Humphrey explained: ‘Only in that way will your dog come to the full realization that it is the responsible guide, it is one to make the decisions and only if it comes to that realization can it make a safe and obedient guide for the sightless master'.48 The dog was never simply being ‘trained’, it had to become ‘responsible’ in a double Harawayian sense: it must learn to understand the threats and obstacles to both itself and a person with visual impairment.

The habitus of a person with visual impairment had to determine the delivery of the instructor’s physical caresses from ‘the first pat you give to the dog’:49

When a blind man pets a dog, he rarely put his hand directly on the dog as he doesn’t know where it is. He moves his hand here and there trying to find the dog and give the pat. In your case, it will be automatic with you (as you caress your dogs with your eyes open) to put your hand directly on the dog when giving a pet. This you must not do. You must endeavour to acquire the groping manner of caress and the groping manner of making each movement that is so characteristic of the sightless. I hope that you will have a sufficient “carry-over” from your blindfold work to be able to do this in your work of caressing and patting your dogs as you did under the blindfold – without the surety of vision.50

Implicit in the passage above is the belief that an instructor’s failure to disconnect their hand movement from their vision endangered the future partnership. With this in mind we can reinterpret caress as constituted not only by the mutual attunement of a dog’s body with that of the instructor, but also with those of the future handler. To further elaborate this, it is worth quoting at length how the requirements of the future handler also demanded that the dog learn to position itself for physical contact and caressing:

You, as a seeing person, may see the dog coming at you, can brace yourself, and, if necessary, get your head out of the way. You, blindfolded or a blind man, would not see the start of the jump and might start to bend over just as the dog jumped so that the dog would hit you, or the blind man with her muzzle.

To avoid this, instructors were advised to caress dogs only ‘while all feet are down’ and never when the dog’s front feet were on the trainer’s body.51

An instructor’s delivery of a verbal caress was determined by the needs and priorities of a visually impaired handler as much as those of the dog and the instructor himself. Take, for example, what appears to a straightforward act: calling the dog by name. An instructor should always articulate the dog’s name in ‘a pleasant and joyful tone’, elaborated Humphrey, as the name ‘must always mean ‘Come’, and coming to master must always be pleasant’.52 Humphrey also exhorted his instructors to never ‘correct it [the dog] while it is away from you and performing the wrong action’ and stressed that ‘[o]nce the dogs comes to you, you must caress it’.53 To ensure a dog would always respond to a handler with visual impairment calling her name, Humphrey made it clear that human emotional self-discipline was essential: ‘You [the instructor] will…have to use your will power to avoid…using the name in angry, corrective tone of voice’. 54 The emotionally undisciplined instructor who failed to submerge their sightedness, to understand the future requirements of the handler with visual impairment would jeopardise the future harmony of the guide dog partnership by projecting themselves into the contact zone, by reducing a dog’s ‘willingness’ to cooperate and collaborate.

While imbuing their name with affective tones provided an important means of satisfying the dog’s emotional requirements, the command also had to anticipate the needs of the visually impaired handler: instructors trained a dog to come to them without hesitation and announce their arrival through physical contact with their left leg:

If you call your dog and it stops away from you, you can see and go to the dog, but a blind man cannot do this as he doesn’t know where his dog is. A blind man whose dog stops away from him might as well not have dog as he cannot find it.

Thus, adding caressing tones to a dog’s name in training helped create a way for a dog to communicate its physical proximity to a handler with visual impairment.

Humphrey’s advice on adjusting the harness further illustrates the necessity of suspending sightedness for the future benefit of the partnership, and required further affective labour. Serving as a prosthesis and interface, the harness facilitated a sensory blending and feedback loop encompassing the handler, the dog and the environment. It constituted a sensitive instrument transmitting non-verbal, corporeal signals, enabling each other’s steps and every variation in speed and change of direction to be communicated and adjusted to. In this way, the harness played an analogous function to the language of caress, helping to constitute an additional shared corporeal language for both parties to learn and respond to each other through as they moved together. Echoing his earlier advice on the initial caress, Humphrey commanded ‘[f]rom the moment you take your harness you must use special care to handle your body as though you were blind’,55 and illustrated this with a striking example to underscore the need to contain the potentially disabling influence of human sight on the affective relationality fostered and facilitated by the harness. When issuing the *Forward* command, Humphrey explained, ‘you can give your gesture in the desired direction with your hand but your body and feet should stay in position until the pull of the harness commands you to move forward’. 56 By stepping off ‘just a split second ahead of your dog’, the instructor had failed to ‘feel’ and respond to the dog’s bodily cues—or ‘commands’—transmitted via the harness. A trained dog who would not start ‘until the man does was an unsafe dog’ as ‘the blind man cannot know when it is safe to start’.57 Using a blindfold, instructed Humphrey, ‘will so accustom you to the feel of the dog that it will be automatic with you to give a *command* and then wait for the movement of the[ir] body [my italics]’. Once the instructor had ‘felt’ their dog’s commands through the harness, they had to follow the dog’s corporeal signals and directions rather than rely on sight for supplementary guidance:

You must remember that to a sightless master, the tension on the harness is the only actual contact between his dog and himself. You and I often neglect that tension because we can see the dog. We can see the surrounding conditions and we follow what we see rather than the tension.58

Thus, according to this understanding, the instructor’s submergence of their sight and acceptance of the dog’s potential agency in the training dyad enabled the dog to act and make a decision independently of the instructor.

As ‘vision gives him confidence’ an instructor’s instinct would be to work as a singular independent entity, rather than within the relationship of dependence and interdependence, thereby hampering the dog’s capacity to act.59 This confidence could lead to working with ‘too light a tension on the harness or irregular tension’, and, when approaching a curb or other obstacle, putting slightly more tension on the harness, causing the dog ‘to check’. Humphrey reasoned that because they were less aware of the environment around them, a handler with visual impairment, dependent on the dog, would exert a greater tension on the harness, and it was imperative this did not imply uncertainty to the dog, causing them to stop and start. Another problem resulting from privileging the sense of sight was a tendency ‘to slow up slightly on approaching a curb or flight of steps’ rather than keeping a consistent pace as the future handler would. This change in speed could become an expected cue and ‘the blind person will find themselves falling over curbs, bumping into obstructions…doing everything…you do not want the blind master to do’.60

Even minute bodily movements, it was thought, could risk undermining a dog’s capacity to make decisions and endanger the interdependent relationship. To illustrate this, Humphrey made a specific point about the tendency of a seeing person to turn their head when in motion to visually take in the environment. Even if this movement was slight, it altered the harness tension and confused the dog. He therefore stipulated a series of bodily adjustments that best approximated those of a handler with visual impairment, including keeping the shoulders square, chin in, and head up and straight:

[i]t simply takes a little practice until you form the habit and the ability of seeing well, not only forward, but to the right and left simply through movements of the eye and not through the movements of the head…It will probably give the impression that you yourself are blind and if you are consistent in working with your head straight ahead.61

An instructor failing to keep his head in a forward, static position, turning to visually take in his environment, risked encouraging the dog to go in the direction his head was turned. ‘The eye of the dog’, remarked Humphrey, ‘is peculiarly sensitive to movement’, perfectly adapted to following a trainer’s gaze and other incidental bodily movements.62 The skill of a dog in responding to even the most subtle of human body movements demanded a radical process of bodily management from the instructor to ensure the safety of the future partnership.

## Multispecies matchmaking

In the final phase the instructor faced a new challenge: partnering the dog they had trained with a carefully selected guide dog owner. The instructor and new handler worked together for a month, at which time the new partnership was tested to ensure they were safe working together. If they passed the test, the new handler was allowed to take the dog home but was required to submit monthly reports on how they were getting along. With the arrival of the visually impaired handler at The Seeing Eye campus, instructors were once again expected to understand and respect the dog’s perceived skill for reading human bodies. The trained dog now found itself with two human handlers, one whose physical contact and verbal caresses they knew intimately, the other a virtual stranger whose caress was unknown: an awkward multispecies party-of-three. The successful and desired outcome of this situation—the transformation of this triad into a harmonious dyad—depended on the trainer’s submission to a particular mode of detachment, facilitating the process of transferring the dog’s affection to the prospective guide dog owner:

[y]ou will see the reason for it if you consider the dog’s point of view. The dog knows the Instructor. It is put with a man whom it doesn’t know and sees no reason for working with this man…There should be as complete a break as possible between the dog and the instructor.63

For Humphrey, the instructor had not merely to stand back during training encounters but self-consciously engage in a sustained deliberate distancing, physically and emotionally, to minimise their capacity to affect their previous canine worker and companion: they must withdraw from the shared language of caress.

To manage the affective processes of detachment from the old handler and attachment to the new, the initial meeting between the guide dog and new handler required careful choreography and could not be left to chance. This process began on campus before the arrival of the prospective guide dog owner through the process of multispecies ‘matchmaking’. Before arriving at the campus and meeting any dogs, the prospective guide dog owner had been matched with a canine partner by the instructors, in accordance with the individual physical capacities, temperaments, personalities and needs of each party.

On arrival at the campus, visually impaired handlers would not meet their dogs for 2 days, during which time they were educated in the rituals of canine courtship and expected to become fluid in the interspecies language of caress, physically and verbally. Furthermore, in the initial encounter with their chosen dog, the new handler had to learn a fundamental lesson about the human–dog relations underpinning The Seeing Eye conception of the guide dog partnership: a dog could not be coerced to do something it did not want to do. When the couple finally met, under close supervision, the new handler was instructed to make the first move by taking ‘a friendly kneeling position’ and letting the dog come to them. Instructors believed ‘[t]he dog will resent any person trying to force its friendships’, and that if the dog was open to the experience of making a new friend, they would move towards the person and activate the relationship.64

The dynamic of detachment/attachment can be illustrated in the days that followed, which were organised in such a way that allowed the dog to respond to the new handler through time and familiarity. With the instructor now in the background, the visually impaired handler and their new dog would sleep in the same room; a significant change for the guide dog, whose previous human companion had never allowed them so close during nocturnal hours, relegating them to a kennel with the other dogs. If the first night went well, the new partners would then spend all day together, without supervision, initiating a further dynamic process of bodily attunement, attachment and detachment. During this time, the visually impaired handler would learn to attend to the dog’s needs, while the dog became accustomed to their new companion’s vocalisations, body language, touch, odour and habits, laying down a crucial foundation on which subsequent training could build.

When the dog and new handler were ready for training, the instructor was reintroduced to teach the pair to walk together. This triadic entity required careful management of affective relationships between all three bodies in order to develop a bond between the new, less experienced handler and the trained dog. In teaching exercises, the instructor had to adhere to a further set of bodily practices to manage the affective potential of his body and prevent the dog looking to him rather than her new partner for cues. Humphrey starkly described the dangers at this time:

Every time you touch or speak to the dog…you simply further the time when the dog is going to pay attention to the new master rather than the instructor. Just so long as you continue to speak to the dog and place or caress the dog, it is going to work for the master it knows better…As long as you touch or speak to the dog, it may be working with the blind master, but is looking past him to you and taking its cues from and working for you. I know this is hard for you to remember, but you must master it. 65

To guard against the instructor accidentally or involuntary communicating cues or affection to their former dog, and thereby jeopardising the cultivated distance, no communication between the trainer and their former canine pupil should take place: ‘All orders, all corrections, all caresses, must [now] come from the blind’. Thus the instructor had to engage in further self-restraint, resisting any urges to give the dog a command, caress or meaningful gesture, and only speaking to the new handler using previously agreed phrases such as ‘thank your dog’ or ‘correct your dog’, which, not being part of the shared language of caress, were unfamiliar to the dog and thus less likely to engage her attention.66

Particular spatial arrangements between the bodies of the training triad were also used to reduce unintentional cues and manage its dynamic relationality. While engaged in harness work out in Morristown, it was imperative that the instructor stood with the new handler between himself and the dog’s line of sight. The body of the instructor was always present but, to enable the dog to act and respond to a new human handler, situated in the background. As the new duo became more attuned, and thus more fluid and precise together, the instructor would distance himself and monitor their performance from afar. However, if something went wrong with the dyad, the instructors were ever present and ready to intervene. The guide dog owner’s intensifying affections for their canine coworker, especially once they both had returned home, could threaten to undo the partnership, and to prevent this demanded further emotional management and diligence from the human side of the partnership. Away from the rationality of the campus, the new handler (now a ‘graduate’) might ‘forget correction when it is needed’ and overlook an ‘offense’, potentially introducing inaccuracies to the dog’s guiding:

The moment you get home, you are going to come to a fuller realization of the value of your dog to you. DO not let this realization cause you to spoil your dog because you love her. We all know parents who spoil their children.67

Unrestrained physical displays of affection and constant play when the dog was not in harness met with disapproval, as the dog would ‘not want to work in harness with you and will become useless to you’.68 A further lesson in the management of canine affections concerned the introduction of the dog to family members and friends, who should be impressed upon ‘to ignore the dog, nor try to play with or caress her’. However, given the intersubjective affective ties between the human–canine partnership, it was imperative the new handler does not get ‘angry’ with their family and friends for behaving in the wrong way: ‘for if you do and speak roughly or with irritation, your dog is going to reflect its master, and at once will get the idea that family and friends are your enemies and hers’.69 The dog should be received into the home ‘not as a pet, nor as a nuisance, but as a fellow worker’.70 Preventing friction in the guide dog partnership and facilitating interdependence thus required cooperation and discipline from other humans. For the sake of harmony and safety within the working partnership, there had to be no doubt in the dog’s mind who was responsible for satisfying their emotional needs.

To a certain extent, this advice given to prospective guide dog owners registers the possibility that the affinities, attunements and connections of embodiment and intercorporeality across species are ever only partial. Guide dog owners were expected to write monthly reports to their instructors to keep them abreast of the difficulties and challenges of attunement when adapting to the working partnership to their home environment and working situations: even when the new partnership left the campus, the new guide dog owner was not left entirely unsupervised, indicative of the limits of attunements of training regimen. In instances where the partnership broke down and the dyad were no longer safe working together, and this included rare instances where they were involved in a traffic accident, retraining was provided for whichever half of the relationship—canine or human—was in need: if further training of the canine half was unsuccessful, the dog would be retired. Thus, the guide dog partnership was never regarded as a finished product; rather it was a living relationship that required concerted effort and monitoring.

## Conclusion

In this article I have examined the material and collective practices developed by The Seeing Eye to produce the guide dog partnership as a case study of dependent agency. By providing a thick description of the ensemble of practices and labour which provided these pioneering visually impaired individuals with the confidence to move through public, urban space with their canine assistants, I have endeavoured to highlight the training of the human partner as much as the canine. The remarkable attention Seeing Eye instructors gave to the interrelated physical, perceptual and emotional interdependence that governed the training of dogs and humans reveals how the guide dog partnership emerged as an epistemological space during the 1930s. Far from being a utilitarian-esque machine, the dog was understood as a living being who, with their human partner, required vigilant cotraining. The Seeing Eye training regimen embraced an explicit self-reflexivity about managing affective relationships of companionship and mutuality, which, it was deemed, was central to securing a successful partnership.

Yet, in making this argument, it is important to acknowledge that The Seeing Eye training manuals present an idealised version of the training process and, moreover, of what a dog could and could not achieve. These are texts laden with specist distinctions and their underlying hierarchies, products of time and place. In her autoethnographic study of well-being in interactions between humans and rescue dogs at an American shelter, Porter sensitively makes a convincing case for interpreting well-being as a trans-species phenomenon in ways that significantly destabilise dog training discourses. In reminding us that the end goal of training is to ‘endanger a dog that can behave in accordance with their adapter’s expectations’, her work raises some uneasy questions ‘about who benefits from certain ways of being and living with other species’.71 While recognising the potentialities of cross-species sociality and connectivity, she provides a more tentative assessment of these prospects and emphasises the need for connections that leave space for the recognition and exploration of partial connections and attunements. From this perspective, while we might consider the guide dog partnership as an exemplary model of interdependence enabling a person with visual impairment to act, the partnership might also be a good place to explore its partial connections, thereby exposing the tensions, stakes and complexities in forging and maintaining interdependent relationships between species.

## Notes

1. Michael Oliver, 1990, *The Politics of Disablement* (Basingstoke: Macmillan Education, 1990).

2. Eva Kittay Kittay, 2011, ”The Ethics of Care, Dependence, and Disability,” *Ratio Juris* 24, no. 1 (2011): 251.

3. Carol Gilligan, 1982, *In a Different Voice: Psychological Theory and Women’s Development* (Cambridge, MA: Harvard University Press, 1982).

4. Dan Goodley et al., 2014, ”Posthuman Disability Studies,” *Subjectivity* 7, no. 4 (2014): 353.

5. Akemi Nishida, 2017, ”Relating through Differences: Disability, Affective Relationality, and the U.S. Public Healthcare Assemblage,” *Subjectivity* 10, no. 1 (2017): 101.

6. Leslie Pickering Francis and Anita Silvers, 2007, “Liberalism and Individually Scripted Ideas of the Good: Meeting the Challenge of Dependent Agency,” *Social Theory and Practice* 33, no. 2 (April 2007): 311–34.

7. Hélène Mialet, 2012, *Stephen Hawking and the Anthropology of the Knowing Subject* (Chicago: Chicago University Press, 2012).

8. Mialet presents Hawking as an exemplar of ‘the distributed centred subject’: he is perceived as the most independent subject—the ‘most powerful’, ‘the most rational’ and the most autonomous (just a brain detached from everything else)—because simultaneously and paradoxically he is also the most connected and most dependent. Mialet, *Stephen Hawking and the Anthropology of the Knowing Subject,* 39–40.

9. Donna Haraway, 2003, *The Companion Species Manifesto: Dogs, People and Significant Others* (Chicago: Prickly Paradigm Press, 2003), 4.

10. Vinciane Despret, 2004, “The Body We Care For: Figures of Anthropo-zoo-geneisis,” *Body and Society* 10, no. 2–3 (2004): 111–34. Despret has explored these ideas across a number of texts, see Vinciane Despret, 2008, “The Becomings of Subjectivity in Animal Worlds,” *Subjectivity* 23, no. 1 (2008): 123–39; Vinciane Despret, 2016, *What Would Animals Say if We Asked the Right Questions?* (Minneapolis and London: University of Minnesota, 2016).

11. Sunaura Taylor, 2017, *Beasts of Burden: Animal and Disability Liberation* (New York and London: The New Press, 2017).

12. Sunaura Taylor, 2017, *Beasts of Burden,* 223.

13. For example, sociologist Clifford Sanders’ description of the guide dog: ‘Most essentially, the dog is a “tool” that functions to assist the visually impaired owner in moving through his or her daily life’. Clifford Sanders (1999), *Understanding Dogs: Living and Working with Canine Companions* (Philadelphia: Temple University Press, 1999), 39.

14. Cary Wolfe, 2010, *What is Posthumanism* (Minneapolis: The University of Minnesota Press, 2010), 141. We might also consider the admiration Andrew Stevenson has expressed for the guide dog partnership for its cross-species sociality, which ‘flouts individuality’, and following Haraway suggestively interprets the ‘dog-guide teams’ as a ‘six-legged’, ‘cross-species’ assemblage, a boundary crossing togethering of human/nonhuman, organic/technological and nature/culture that ‘define each other and conjure worlds they move through’. Andrew Stevenson (2013), “Dog Team Walking: Inter-Corporeal Identities, Blindness and Reciprocal Guiding,” *Disability and Society* 28, no. 8 (2013): 1164.

15. Philip Howell, 2018, “Animals, Agency and History” in *The Routledge Companion to Animal-Human History*, eds. Philip Howell and Hilda Kean (London and New York: Routledge, 2018), 197–221.

16. On the history of other guide dog schools, see Monika Baár (2015), “Disability and Civic Courage under State Socialism: The Scandal over the Hungarian Guide-Dog School,” *Past and Present* 227, no. 1 (2015): 179–203; Monika Baár (2015), “Prosthesis for the Body and for the Soul: the Origins of Guide Dog Provision for Blind Veterans in Interwar Germany,” *First World War Studies* 6, no. 1 (2015): 81–98; Julie Anderson and Neil Pemberton (2007), “Walking Alone: Aiding the War and Civilian Blind in the Interwar Period,” *European Review of History* 14, no. 4 (2007): 459–79.

17. Justyna Wlodarczyk, 2018, *Genealogy of Obedience: Reading North American Pet Dog Training Literature, 1850s-2000s* (Leiden: Brill, 2018). For a history of science approach see Robert G W Kirk (2014), “In Dog’s We Trust? Intersubjectivity, Response-Able Relations and the Making of Mine Detector Dogs,” *Journal of the History of the Behavioural Sciences* 50, no. 1 (2014): 1–36.

18. Chris Pearson, 2016, “Between Instinct and Intelligence: Harnessing Police Dog Agency in Early Twentieth-Century Paris,” *Comparative Studies in Society and History* 58, no. 2 (2016): 463–490. https://doi.org/10.1017/S0010417516000141.

19. Peta Tait, 2012, *Wild and Dangerous Performances: Animals, Emotions, Circus* (Basingstoke: Palgrave, 2012).

20. Natalie Hannah Porter, 2018, “Training Dogs to Feel Good: Embodying Well-being in Multispecies Relations,” *Medical Anthropology Quarterly*. https://doi.org/10.1111/maq.12459.

21. Historical dimensions of canine–human companionship relationships have been explored in some depth, see Philip Howell, 2015, *At Home And Stray: The Domestic Dog in Victorian Britain* (Virginia: University of Virginia, 2015).

22. The Seeing Eye Archives. Jack Humphrey Hubbard, Lectures. 20 Volumes. They are referred to as the Hubbard Lectures because the transcription was financed by Helen Hubbard, a patron of The Seeing Eye.

23. On war as a catalyst to new conceptualisations of disability see Julie Anderson (2011), *War, Disability and Rehabilitation: The Soul of the Nation* (Manchester: Manchester University Press, 2011); Ana Carden-Coyne (2009), *Reconstructing the Body: Classicism, Modernism and the First World War* (Oxford: Oxford University Press, 2009); John Matthew Kinder, *Paying with their Bodies: American War and the Problem of the Disabled Veteran* (Chicago: Chicago University Press, 2015); Heather Perry (2014), *Recycling the Disabled: Army, Medicine and Modernity in WWI Germany* (Manchester: Manchester University Press, 2014); Beth Linker (2011), *War’s Waste: Rehabilitation in World War I America* (Chicago: Chicago University Press, 2011); Jeffrey Reznick (2011), *The Culture of Caregiving* (Manchester: Manchester University Press, 2011).

24. Monika Baár, 2015, “Prosthesis for the Body and for the Soul,” 81–98. Though, even if we accept this to be the case, as Chris Pearson has argued, it was not without serious tensions and contradictions, as shown by Chris Pearson in his examination of the tensions in mobilising the canine–human bond in the context of the First World War. Chris Pearson, “‘Four-Legged Poilus’: French Army Dogs, Emotional Practices and the Creation of Militarised Human-Dog Bonds, 1870–1918,” *Journal of Social History* 52, no. 3 (2019). https://doi.org/10.1093/jsh/shx090.

25. Although its transnational ambitions to create guide dog schools waned in the wake of the Great Depression, The Seeing Eye helped to establish guide dog schools in Britain, Italy and France. This origins of The Seeing Eye is well known and a number of accounts focus on the pioneers, especially Dorothy Eustis. Miriam Ascarelli (2010), *Dorothy Harrison Eustis and the Story of The Seeing Eye* (West Lafayette: Purdue University Press, 2010); Peter Brock Putnam (1979), *Love in the Lead: The Fifty-Year Miracle of the Seeing Eye Dog* (New York: Dutton, 1979).

26. Arthur C Bartlett, 1933, *Skipper, the Guide Dog* (Boston: WA Wilde Company, 1933), 6.

27. Paul K. Longmore and David Goldberger, 2000, “The League of the Physically Handicapped and the Great Depression: A Case Study in the New Disability History,” *The Journal of American History* 87, no. 5 (2000): 888–922.

28. Justyna Wlodarczyk, 2018, *Genealogy of Obedience,* 80–105.

29. As Justyna Wlodarczyk (2018) has observed, breeding and kennelling the German shepherd was rooted in both shifts in the perceived canine usefulness in the service of the state, but also they were embedded in broader understandings of man–nature relationships and white masculinity.

30. Anonymous, 1935, “Hundreds of Blind Seek Chance to get Dog Guides of Seeing Eye,” *New York Herald Tribune*, April 4, 1935.

31. Humphrey, Hubbard Lectures, vol. 2, 159.

32. Humphrey, Hubbard Lectures, vol. 2, 159.

33. Humphrey, Hubbard Lectures, vol 1, 25–27.

34. Humphrey, Hubbard Lectures, vol. 2, 149.

35. Humphrey, Hubbard Lectures, vol. 2, 145–48.

36. Humphrey, Hubbard Lectures, vol. 15, 1598.

37. Thomas Dixon, 2003, *From Passions to Emotions: The Creation of a Secular Psychological Category* (Cambridge: Cambridge University Press, 2003).

38. Humphrey, Hubbard Lectures, vol. 15, 1598.

39. Despret, “The Body We Care For,” 134.

40. Humphrey, Hubbard Lectures, vol.2, 149.

41. Humphrey, Hubbard Lectures, vol. 2, 149.

42. Humphrey, Hubbard Lectures, vol. 16, 1778.

43. Humphrey, Hubbard Lectures, vol. 12, 1178.

44. Humphrey, Hubbard Lectures, vol. 7, 907.

45. Humphrey, Hubbard Lectures, vol. 1, 23.

46. Humphrey, Hubbard Lectures, vol. 9, 832.

47. Humphrey, Hubbard Lectures, vol. 9, 832.

48. Humphrey, Hubbard Lectures, vol. 7, 572.

49. Humphrey, Hubbard Lectures, vol. 2, 164.

50. Humphrey, Hubbard Lectures, vol. 2, 164.

51. Humphrey, Hubbard Lectures, vol. 1, 26.

52. Humphrey, Hubbard Lectures, vol. 2, 100.

53. Humphrey, Hubbard Lectures, vol. 2, 100.

54. Humphrey, Hubbard Lectures, vol. 2, 168.

55. Humphrey, Hubbard Lectures, vol 3, 240.

56. Humphrey, Hubbard Lectures, vol 2, 118.

57. Humphrey, Hubbard Lectures, vol: 2, 119

58. Humphrey, Hubbard Lectures, vol. 2, 109.

59. Humphrey, Hubbard Lectures, vol. 4, 380.

60. Humphrey, Hubbard Lectures, vol. 2, 259.

61. ‘That might embarrass you but I am going to tell you now that from my standpoint no greater compliment can be paid to an instructor than for people to think he is blind’. Humphrey, Hubbard Lectures, vol. 2, 250.

62. Humphrey, Hubbard Lectures, vol. 12, 1139.

63. Humphrey, Hubbard Lectures, vol. 6, 582.

64. Humphrey, Hubbard Lectures, vol. 7, 628.

65. Humphrey, Hubbard Lectures, vol. 6, 563.

66. Humphrey, Hubbard Lectures, vol. 4, 403.

67. Humphrey, Hubbard Lectures, vol. 6, 679.

68. Humphrey, Hubbard Lectures, vol. 6, 699.

69. Humphrey, Hubbard Lectures, vol. 7, 824.

70. Humphrey, Hubbard Lectures, vol. 7, 824.

71. Natalie Hannah Porter, 2018, “Training Dogs to Feel Good,” 6.
